# Monitoring measurable residual disease in paediatric acute lymphoblastic leukaemia using immunoglobulin gene clonality based on next-generation sequencing

**DOI:** 10.1186/s12935-024-03404-3

**Published:** 2024-06-25

**Authors:** Won Kee Ahn, Kyunghee Yu, Hongkyung Kim, Seung-Tae Lee, Jong Rak Choi, Jung Woo Han, Chuhl Joo Lyu, Seungmin Hahn, Saeam Shin

**Affiliations:** 1 Division of Pediatric Hematology and Oncology, Department of Pediatrics, Severance Hospital, Yonsei Cancer Center, Yonsei University College of Medicine, 50-1 Yonsei-ro, Seodaemun-gu, Seoul, 03722 Republic of Korea; 2 Department of Laboratory Medicine, Severance Hospital, Yonsei University College of Medicine, 50-1 Yonsei-ro, Seodaemun-gu, Seoul, 03722 Republic of Korea; 3 Department of Laboratory Medicine, Chung-Ang University College of Medicine, Seoul, Republic of Korea; 4 Dxome Co. Ltd, Seongnam-si, Gyeonggi-do, Korea

**Keywords:** Acute lymphoblastic leukaemia, Immunoglobulin heavy chain genes, Immunoglobulin kappa light chain genes, Measurable residual disease, Next-generation sequencing

## Abstract

**Background:**

Assessment of measurable residual disease (MRD) is an essential prognostic tool for B-lymphoblastic leukaemia (B-ALL). In this study, we evaluated the utility of next-generation sequencing (NGS)–based MRD assessment in real-world clinical practice.

**Method:**

The study included 93 paediatric patients with B-ALL treated at our institution between January 2017 and June 2022. Clonality for *IGH* or *IGK* rearrangements was identified in most bone marrow samples (91/93, 97.8%) obtained at diagnosis.

**Results:**

In 421 monitoring samples, concordance was 74.8% between NGS and multiparameter flow cytometry and 70.7% between NGS and reverse transcription-PCR. Elevated quantities of clones of *IGH* alone (*P* < 0.001; hazard ratio [HR], 22.2; 95% confidence interval [CI], 7.1–69.1), *IGK* alone (*P* = 0.011; HR, 5.8; 95% CI, 1.5–22.5), and *IGH* or *IGK* (*P* < 0.001; HR, 7.2; 95% CI, 2.6–20.0) were associated with an increased risk of relapse. Detection of new clone(s) in NGS was also associated with inferior relapse-free survival (*P* < 0.001; HR, 18.1; 95% CI, 3.0–108.6). Multivariable analysis confirmed age at diagnosis, *BCR::ABL1*-like mutation, *TCF3::PBX1* mutation, and increased quantity of *IGH* or *IGK* clones during monitoring as unfavourable factors.

**Conclusion:**

In conclusion, this study highlights the usefulness of NGS-based MRD as a routine assessment tool for prognostication of paediatric patients with B-ALL.

**Supplementary Information:**

The online version contains supplementary material available at 10.1186/s12935-024-03404-3.

## Introduction

Acute lymphoblastic leukaemia (ALL) is the most common malignancy in childhood. Approximately 80–85% of cases are of B-cell lineage. B-lymphoblastic leukaemia (B-ALL) in children usually has a favourable prognosis, with an overall complete remission rate of > 95% [1, 2]. However, 15–20% of patients experience relapse, one of the most common causes of treatment failure in paediatric ALL [3]. Measurable residual disease (MRD) refers to the persistence of leukaemic cells below the threshold of conventional morphologic assessment. The presence of MRD throughout sequential therapy is associated with an increased risk of relapse and adverse outcomes in patients with ALL [4–6]. Therefore, MRD monitoring has become a routine clinical practice in treating B-ALL.

Highly sensitive and specific techniques are required when monitoring MRD. Multiparameter flow cytometry (MFC) to track aberrant immunophenotypes is widely used to quantify MRD in ALL because of its quick turnaround time and broad applicability in many patients. However, it has technical limitations, including difficulties with standardising antibody panels, gating strategies, and number of analysed cells across laboratories, resulting in variable sensitivity [7, 8]. Moreover, a high level of expertise is required to accurately interpret the results, and phenotypic shifts in antigen expression of leukaemic B-lymphoblasts may occur after chemotherapy [7, 9]. Real-time quantitative polymerase chain reaction (PCR) is highly sensitive for detecting target fusion transcripts, such as *BCR::ABL1*, but is applicable for only 30–40% of patients. Allele-specific oligonucleotide PCR targeting immunoglobulin genes is another traditional method of tracking MRD but requires customised patient-specific primers and individual optimisation of testing conditions. Although it has high sensitivity and broad applicability in MRD detection, it is not routinely used in many clinical laboratories because of its burdensome and time-consuming nature [9].

Next-generation sequencing (NGS) for tracking clonal immunoglobulin and T-cell receptor gene rearrangements is an emerging method of MRD assessment [9, 10]. NGS-based MRD assay uses consensus primers that universally amplify rearranged immunoglobulin gene segments. Therefore, it does not require patient-specific customisation and can detect clonal evolution during treatment. Many studies confirmed its high sensitivity, reliability in clinical settings, and universal applicability [11–16]. In patients with B-ALL who achieved MRD negativity after induction chemotherapy, NGS detected positive MRD conversion earlier than MFC, with conversion being detected a median of 4.7 months before clinical relapse [10]. Moreover, the prognostic significance of NGS-based MRD status during and after treatment has been established in several studies, especially in children [7, 11–13].

In this study, we analysed clonal rearrangements of immunoglobulin heavy chain (*IGH*) and immunoglobulin kappa light chain (*IGK*) in paediatric patients with B-ALL. We explored MRD data obtained via MFC and NGS methods and evaluated clinical aspects and differences in prognosis based on MRD status.

## Methods

### Patients and study design

Between January 1, 2017 and June 30, 2022, we collected 430 bone marrow samples from 93 paediatric patients diagnosed with B-ALL, including those with newly diagnosed and relapsed disease. Initial bone marrow assessments were performed using flow cytometry, genetic studies and NGS clonality assays. All patients were classified according to the World Health Organization classification, 5th edition. Risk group was determined using the National Cancer Institute (NCI) criteria and re-stratified after the induction treatment according to the classification of their genetic mutations and treatment outcomes. Treatment was administered based on the risk-stratified treatment protocol at Yonsei Cancer Center, Yonsei University Health System, Seoul, Korea (Figure S1). Bone marrow biopsies were performed using flow cytometry, and MRD assays were conducted using PCR or NGS clonality, depending on the specified targets for each time period. The study protocol was approved by the Institutional Review Board (IRB) of the Yonsei University Health System Clinical Trials Center (IRB 4-2021-0090).

### Cytogenetic and molecular genetic analysis

Conventional G-banding karyotyping was performed on bone marrow aspirates according to standard protocols. Fluorescence in situ hybridisation (FISH) was performed using the *IGH/MYC* and *BCR/ABL* dual fusion probe, *CDKN2A/CEP9* (p16) dual spot probe, *ETV6*(*TEL*)/*RUNX1*(*AML1*) ES dual colour extra signal probe, and *MLL* break apart probe (Abbott Vysis, Des Plaines, IL). Recurrent translocations were identified by reverse transcription-PCR (RT-PCR) using a HemaVision kit (DNA Technology, Aarhus, Denmark) or NGS RNA panel testing using a FusionPlex Pan-Heme Kit (ArcherDx, Boulder, CO, USA). Targeted NGS panel testing of genomic DNA was performed using custom probes (Dxome Co. Ltd., Gyeonggi-do, Korea) targeting 185 genes or 497 genes recurrently mutated in haematologic malignancies. Data analysis for detecting single nucleotide variants and copy number variants was conducted as previously described [17].

### MRD monitoring using LymphoTrack assay

To screen and monitor clonal *IGH* and *IGK* rearrangements at diagnosis and during follow-up, we performed NGS clonality analysis using the LymphoTrack® *IGH* FR1 Assay and LymphoTrack® *IGK* Assay (InVivoScribe Technologies, San Diego, CA, USA). Genomic DNA was extracted using the QIAsymphony DNA Mini Kit (Qiagen, Hilden, Germany), according to the manufacturer’s recommendations, and then quantified using the Qubit® 2.0 Fluorometer (Invitrogen, Carlsbad, CA). Genomic DNA (median 250 ng, range 69 − 11,040) was amplified using a single multiplex master mix for each target. Amplicons were subsequently purified using the Agencourt®AMPure XP system (Beckman Coulter, Brea, CA) and quantified using the Agilent 2100 BioAnalyzer (Agilent Technologies, Inc., Santa Clara, CA). Libraries were sequenced on the MiSeq Dx system using the Miseq Reagent Kit v2 (Illumina, San Diego, CA). Raw FASTQ files were analysed using LymphoTrack Software–MiSeq v2.4.3 (InVivoScribe Technologies). The cutoffs for clonotype determination followed the manufacturer’s guidelines. For MRD monitoring, the initial clonotype sequence was tracked using LymphoTrack MRD Software 2.0.2. MRD level was calculated as recommended by the manufacturer: sequence of the initial clonotype sequence (exact match read count plus 1 and 2 mismatch read counts) divided by the total number of reads generated by the sample. The median (range) sequencing reads included in our study were 308,499 (25,564 − 14,324,828). According to the manufacturer, sequencing 300 ng of DNA with 250,000 reads guarantees a sensitivity level of 10^− 4^ with approximately 95% confidence. We defined MRD positivity as > 1 × 10^− 4^, based on the assay’s sensitivity. Additionally, we tracked elevated MRD levels, indicating increases from previous measurements over time in each patient.

### MRD monitoring using flow cytometry and RT-PCR

MFC was performed at diagnosis with three 8-colour antibody combinations: CD38-FITC/cMPO (cytoplasmic myeloperoxidase)-PE/CD117-PerCPCy5.5/CD34-PECy7/cCD79a-APC/CD45-APC750/CD19-PB/cCD3-OC515, TdT (terminal deoxynucleotidyl transferase)/CD20/CD13/CD34/cCD22/CD10/CD19/CD45, and CD7/CD11c/CD33/CD34/CD117/CD14/HLA-DR/CD45. Data were analysed using the Beckman Coulter Navios instrument and Kaluza software (Beckman Coulter, Miami, FL, USA). For MRD analysis, we used a combination of markers identified at diagnosis. At least 5 × 10^5^ events were acquired, and the limit of quantification was 0.01%.

For RT-PCR, total RNA was extracted from bone marrow aspirates or whole blood using the QIAamp RNA Blood Mini Kit (Qiagen), according to the manufacturer’s instructions. RT-PCR for *BCR::ABL1* transcript monitoring was performed with the ipsogen® BCR-ABL1 Mbcr Kit (Qiagen) and ABI 7500 Real-Time PCR System (Thermo Fisher Scientific, Waltham, MA).

### Treatment

The patients’ treatment was based on their NCI risk group. Standard-risk (SR) patients (aged > 365 days to < 10 years and leukocyte count < 50,000/µL at diagnosis) received a 3-drug regimen during a 4-week induction phase and were re-stratified at the end of induction according to their genetic mutations and treatment response on bone marrow biopsy. High-risk (HR) patients received a 4-drug regimen during both induction and consolidation (4 weeks for each phase) and were then re-stratified according to MRD status (Figure S1).

Maintenance therapy or haematopoietic stem cell transplantation (HSCT) was based on treatment response and patient risk factors. Patients with *BCR::ABL1* mutation received a BCR-ABL tyrosine kinase inhibitor (imatinib or dasatinib) as part of their treatment. HSCT was recommended for patients with sustained MRD positivity and high-risk genetic features, such as hypoploidy, *BCR::ABL1* rearrangement, or *BCR::ABL1*-like mutations. Bone marrow biopsies were obtained at diagnosis, after induction and consolidation chemotherapy, 2 additional times before maintenance (Figure S1).

### Statistical analysis

Data are presented as median with interquartile range (IQR), number with percentage, and mean ± standard deviation. To explore the relationship between NGS clonality and other parameters, we determined Pearson correlation coefficients.

The primary study outcomes were relapse-free survival (RFS) and overall survival (OS). We also examined whether NGS clonality was a predictor of disease prognosis. Survival duration was calculated from the date of diagnosis to the last follow-up date. RFS was defined as the time from diagnosis to the time of first relapse or progressive disease; and OS as the time from diagnosis to death from any cause. Cox regression analysis was performed to estimate hazard ratios and 95% confidence intervals (CIs), and *P*-values were determined to compare distributions of outcomes. Statistical significance was set at *P*-values < 0.05. All analyses were conducted using R statistical software version 4.2.2 (Foundation for Statistical Computing, Vienna, Austria).

## Results

### Patient and disease characteristics

Of the 93 patients enrolled in this study, 51 were assigned to the SR group and 42 to the HR group. Patient and disease characteristics are summarised in Table 1. After the induction phase, 12 patients were reclassified according to remission status and genetic mutation, with 10 SR patients (19.6%) transitioning to the HR group and 2 h patients reassigned to the SR group. Remission was not achieved in 1 h group patient (2.4%). Fifteen patients (16.1%) underwent HSCT. Median follow-up duration was 2.59 years (IQR, 1.36–3.67).

**Table 1:** Patient and disease characteristics

Characteristic		SR group (*n* = 51)		HR group (*n* = 42)		Total cohort (*N* = 93)	
		No.	Percentage	No.	Percentage	No.	Percentage
**Sex**	Male	27	52.9%	23	54.8%	50	53.8%
	Female	24	47.1%	19	45.2%	43	46.2%
**Age at diagnosis, y** (median, IQR)		3.55	2.88–5.33	13.04	10.48–15.07	6.12	3.14–12.52
**Blast % at diagnosis** ^**a**^ (median, IQR)		81.2%	55.8–89.5	83.6%	68.6–92.8	83.2%	61.0–92.1
**WBC count at diagnosis** ^**b**^ (median, IQR)		5,130	3,040–16,490	11,670	3,535–447,500	7,540	3,170–26,120
**Genetic subtype**	Normal karyotype	7	13.7%	8	19.0%	15	16.1%
	Other	3	5.9%	8	19.0%	11	11.8%
	*ETV6::RUNX1* rearrangement	15	29.4%	5	11.9%	20	21.5%
	Hyperdiploidy	23	45.1%	7	16.7%	30	32.3%
	*BCR::ABL1* rearrangement	0	0.0%	5	11.9%	5	5.4%
	*BCR::ABL1*-like	2	3.9%	4	9.5%	6	6.5%
	*TCF3::PBX1* rearrangement	0	0.0%	3	7.1%	3	3.2%
	iAMP21	1	2.0%	0	0.0%	1	1.1%
	*KMT2A* rearrangements	0	0.0%	2	4.8%	2	2.2%
**Immunoglobulin gene rearrangement**	*IGH* FR1 clonality positive	49	96.1%	38	90.5%	87	93.5%
	*IGK* clonality positive	33	64.7%	28	66.7%	61	65.6%
	*IGH* FR1 and *IGK* clonality positive	33	64.7%	33	78.6%	56	60.2%
	*IGH* FR1 and *IGK* clonality both negative	2	3.9%	0	0.0%	2	2.2%
**Reclassification of risk group**		10	19.6%	2	4.8%	12	12.9%
**HSCT**		4	7.8%	11	26.2%	15	16.1%

^a^ On bone marrow biopsy

^b^ In peripheral blood

Abbreviations: BM, bone marrow; HR, high-risk; HSCT, haematopoietic stem cell transplantation; IQR, interquartile range; SR, standard-risk; WBC, white blood cell

**Fig. 1:**
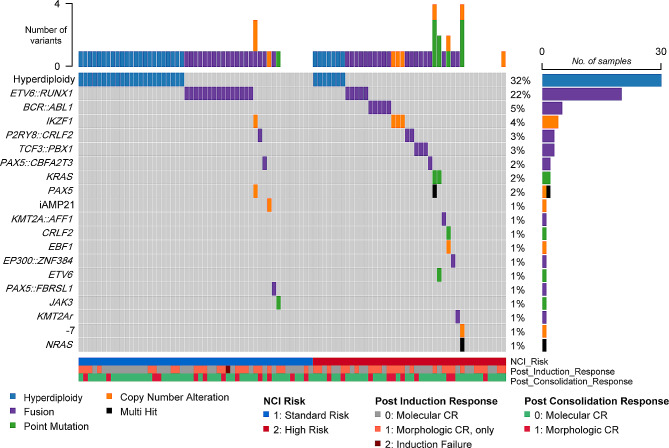
Spectrum of genetic subtypes identified in bone marrow samples of patients at the time of diagnosis, stratified by National Cancer Institute (NCI) risk and treatment response. Abbreviation: CR, complete remission

Figure 1 shows the genetic subtypes identified in bone marrow samples at diagnosis. Hyperdiploidy and *ETV6::RUNX1* were the most commonly detected genetic alterations, followed by *BCR::ABL1*, *IKZF1* mutation, *P2RY8::CRLF2*, and *TCF3::PBX1*. The genetic subtypes were stratified by NCI risk, post-induction response, and post-consolidation response. Hyperdiploidy and *ETV6::RUNX1* rearrangement were more prevalent in the SR group, whereas *BCR::ABL1* and *KMT2A* rearrangements were present only in the HR group.

### LymphoTrack result

Index clonal sequences were established in 91 patients (97.8%), with most patients (93.5%) exhibiting positivity in *IGH* FR1. Only 2 patients exhibited no detectable clonal sequences in the LymphoTrack assay. Among the 58 patients lacking target markers for FISH or RT-PCR testing, 54 (93.1%) tested positive in the LymphoTrack assay.

The distributions of *IGH* and *IGK* clones are shown in Figure S2. For *IGH*, the most prevalent pattern was the presence of 2 clones (36.6%), followed by 1 clone (26.9%), 3 clones (17.2%), and 4–6 clones (12.9%). For *IGK*, the most prevalent pattern was 1 clone (28.0%), followed by 2 clones (20.4%) and 3–6 clones (18.3%).

The *BCR::ABL1*-like mutation exhibited an odds ratio of 11.31 (*p* < 0.01) for positive MRD. However, the other characteristics and genetic mutations showed no association with the sustained MRD state in bone marrow sampling.

### Treatment response

In total, 92 patients (98.9%) achieved morphologic complete remission (CR). Among 91 patients with a positive LymphoTrack assay, 49 (53.8%) did not achieve molecular CR in *IGH* or *IGK* after the induction phase (Table 2). After the consolidation phase, 22 patients (24.2%) did not achieve molecular CR, and 16 patients (17.6%) experienced an increase of MRD level in LymphoTrack, compared to the end of induction. Figure 1 summarises baseline ALL characteristics and treatment responses according to morphologic or molecular CR.

**Table 2:** Response to chemotherapy at the end of induction and end of consolidation

Time	Outcome	SR group			HR group			Total cohort		
		No.	Total	Percentage	No.	Total	Percentage	No.	Total	Percentage
**EOI**	CR	50	51	98.0%	42	42	100%	92	93	98.9%
	MFC CR	48	51	94.1%	39	42	92.9%	87	93	93.5%
	*IGH* MRD (> 10^− 4^)^a^	20	49	40.8%	14	38	36.8%	34	87	39.1%
	*IGK* MRD (> 10^− 4^)^b^	13	33	39.4%	15	28	53.6%	28	61	45.9%
	*IGH* or *IGK* MRD (> 10^− 4^)^c^	25	49	51.0%	24	42	57.1%	49	91	53.8%
**EOC**	Morphologic CR	51	51	100%	42	42	100%	93	93	100%
	MFC CR	45	51	88.2%	37	42	88.1%	82	93	88.2%
	*IGH* MRD (> 10^− 4^)^a^	6	49	12.2%	8	38	21.1%	14	87	16.1%
	*IGK* MRD (> 10^− 4^)^b^	7	33	21.2%	5	28	17.9%	12	61	19.7%
	*IGH* or *IGK* MRD (> 10^− 4^)^c^	12	49	24.5%	10	42	23.8%	22	91	24.2%
	Elevation of clonality^d^	10	49	20.4%	6	42	14.3%	16	91	17.6%

^a^ Performed on patients with positive *IGH* clonality at the time of diagnosis

^b^ Performed on patients with positive *IGK* clonality at the time of diagnosis

^c^ Performed on patients with positive *IGH* or *IGK* clonality at the time of diagnosis

^d^ Elevation of clonality of *IGH* or *IGK*, compared with the clonality at the end of induction

Abbreviations: CR, complete remission; EOC, end of consolidation for high-risk patients and end of first interim-maintenance for standard-risk patients; EOI, end of induction; HR, high-risk; MFC, multiparameter flow cytometry; MRD, measurable residual disease; SR, standard-risk

### Concordance between LymphoTrack and flow cytometry or RT-PCR

A total of 421 monitoring samples of the 91 patients with index clonal sequences were analysed during treatment. Tumour burden was positively correlated with the LymphoTrack assay. Pearson correlation coefficients were 0.81 between the percentage of blast cells and *IGH* clonality and 0.79 between the percentage of blast cells and *IGK* clonality (Figure S3).

Table 3 displays concordance rates between LymphoTrack and flow cytometry or RT-PCR. Concordance was observed between LymphoTrack and flow cytometry in 74.8% of samples (247/330) and between LymphoTrack and RT-PCR in 70.7% of samples (70/99).

**Table 3:** Concordance of measurable residual disease status between next-generation sequencing clonality and multiparametric flow cytometry or reverse transcription-polymerase chain reaction analysis

MFC		EOI	EOC	First f/u	Second f/u	Total	Percentage	Rate
		(*n* = 91)	(*n* = 91)	(*n* = 77)	(*n* = 71)			
Concordant								74.8%
	MFC+/NGS+	5	8	3	2	18	5.5%	
	MFC-/NGS-	41	67	63	58	229	69.4%	
Discordant								25.2%
	MFC-/NGS+	44	14	10	10	78	23.6%	
	MFC+/NGS-	1	2	1	1	5	1.5%	
Total		91	91	77	71	330	100%	-
**RT-PCR**		**EOI**	**EOC**	**First f/u**	**Second f/u**	**Total**	**Percentage**	**Rate**
		**(** *n* ** = 30)**	**(** *n* ** = 28)**	**(** *n* ** = 23)**	**(** *n* ** = 20)**			
Concordant								70.7%
	RT-PCR+/NGS+	5	3	1	1	10	10.1%	
	RT-PCR-/NGS-	12	19	15	14	60	60.6%	
Discordant								29.3%
	RT-PCR-/NGS+	12	5	4	4	25	25.3%	
	RT-PCR+/NGS-	1	1	1	1	4	4.0%	
Total		30	28	21	20	99	100%	-

Abbreviations: EOC, end of consolidation for high-risk patients and end of first interim-maintenance for standard-risk patients; EOI, end of induction; f/u, follow-up; MFC, multiparametric flow cytometry; MRD, measurable residual disease; NGS, next-generation sequencing; RT-PCR, reverse transcription-polymerase chain reaction

### Survival outcomes

For the entire study cohort, the 3-year RFS and OS rates were 93.5% (95% CI, 87.5–100%) and 93.3% (95% CI, 87.8–99.2%), respectively. The 3-year RFS was 100% in the SR group and 86.0% (95% CI, 73.8–100%) in the HR group. The 3-year OS was 94.9% (95% CI, 88.3–100%) in the SR group and 91.8% (95% CI, 83.4–100%) in the HR group. Outcomes were inferior for patients with MRD positivity on the LymphoTrack assay, particularly for *IGH*, compared to those with no MRD positivity (Figure S4). Five patients (5.4%) died: 4 from treatment-related complications (sepsis and pneumonia) and 1 from an accident. There were no mortalities directly related to disease progression.

**Fig. 2:**
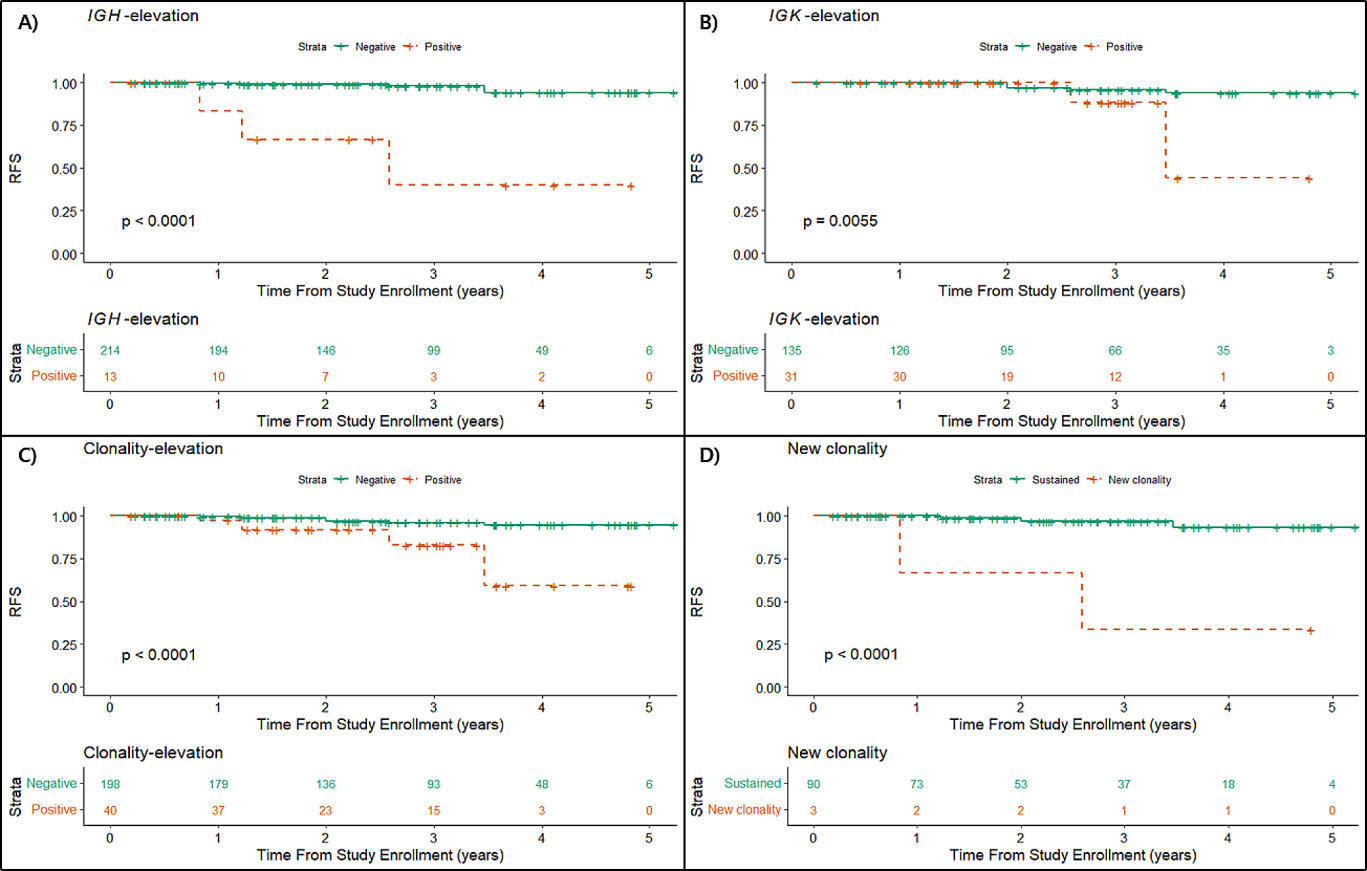
Relationships between changes in clonality and relapse-free survival (RFS). In this analysis, patients who relapsed more than once were removed to avoid duplication. Patients who experienced an increase in quantities of clones of *IGH* alone (A), *IGK* alone (B), and *IGH* or *IGK* (C) had a higher risk of relapse. Emergence of a new clone was also associated with a higher risk of relapse, compared to no emergence of a new clone (D)

We also examined outcomes related to increases in NGS clonality over time. All patients with an increase in quantity of clones or conversion to positive NGS clonality, exhibited inferior RFS, compared to patients with sustained negativity or decreased NGS clonality (Fig. 2). Elevated MRD levels of *IGH* alone (*P* < 0.001; hazard ratio, 22.2; 95% CI, 7.1–69.1), *IGK* alone (*P* = 0.011; hazard ratio, 5.8; 95% CI, 1.5–22.5), and *IGH* or *IGK* (*P* < 0.001; hazard ratio, 7.2; 95% CI, 2.6–20.0) were significantly associated with increased risk of relapse. Detection of new clone(s) on NGS clonal assay was also associated with inferior RFS (*P* < 0.001; hazard ratio, 18.1; 95% CI, 3.0–108.6).

Univariable Cox proportional hazards regression analysis identified age at diagnosis, *TCF3::PBX1* mutation, elevated MRD levels of *IGH* clones alone during monitoring, elevated MRD levels of *IGH* or *IGK* clones during monitoring, and emergence of new clone(s) as significant unfavourable factors for RFS (Table S1). Multivariable Cox regression confirmed age at diagnosis, *BCR::ABL1*-like mutation, *TCF3::PBX1* mutation, and elevated *IGH* or *IGK* clone quantity as significant unfavourable factors.

### Relapse

**Fig. 3:**
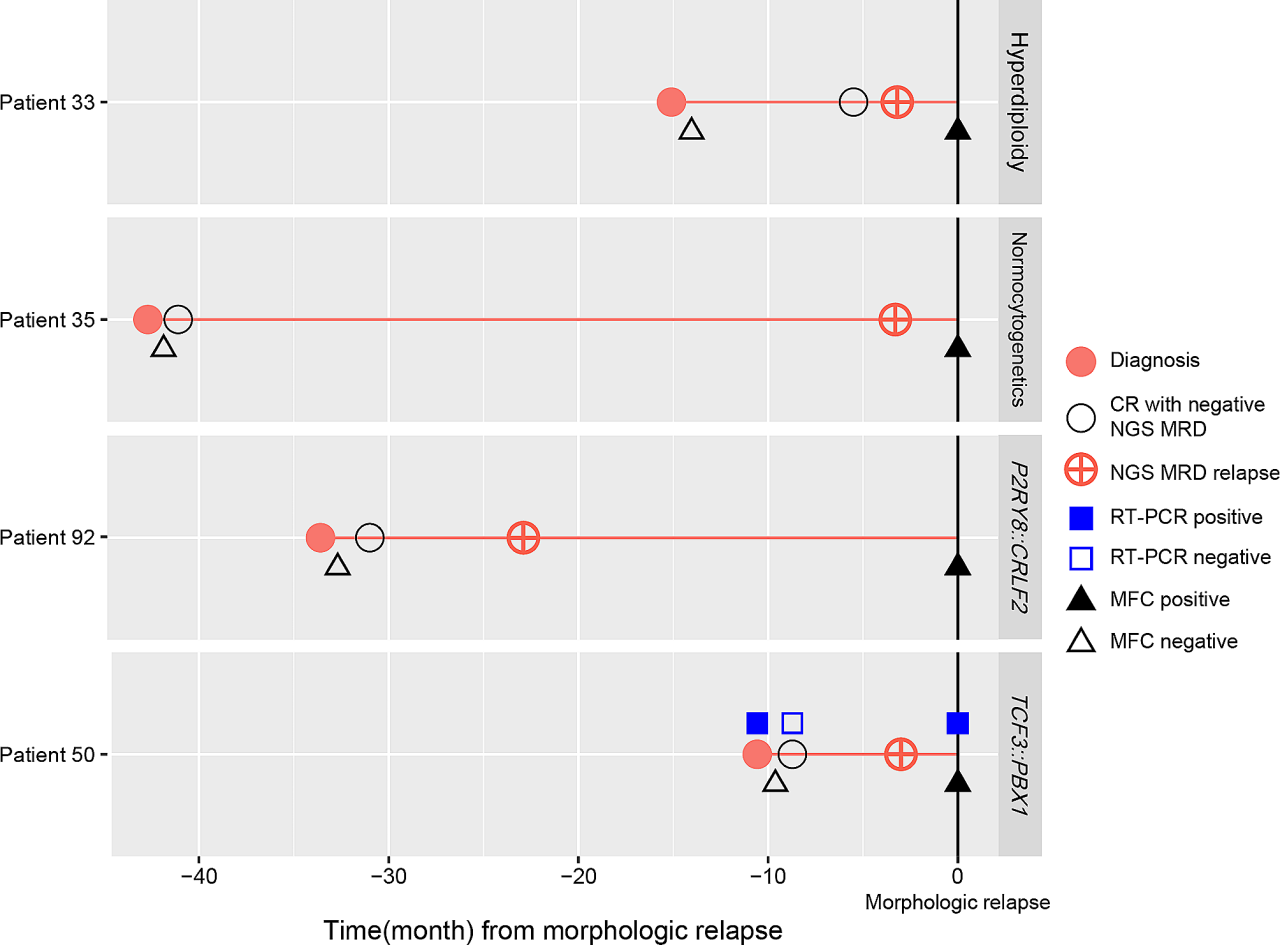
Swimmer plot showing disease monitoring status in 4 patients with bone marrow relapse. Measurable residual disease (MRD) status based on next-generation sequencing (NGS) clonality, reverse transcription-polymerase chain reaction (RT-PCR) and Multiparameter flow cytometry (MFC) are shown. The patients had different genetic subtypes at the time of diagnosis (indicated on the right side of the figure). Abbreviation: CR, complete remission

Five (5.4%) patients experienced relapse, which was extramedullary in 1 patient and involved the bone marrow in 4 patients (Fig. 3, Figure S5). All patients with bone marrow relapse experienced increases in the clonality assay before morphologic relapse. Median duration between detection of clonality elevation and morphologic relapse was 95.33 days (range, 87.29–1007.13). In 2 patients, new clones emerged prior to the morphologic relapse.

## Discussion

Assessment of MRD status throughout treatment, as well as during the post-treatment phase, has well-established prognostic significance in paediatric B-ALL [4–6]. In this study, we conducted a comprehensive examination of MRD data acquired using the LymphoTrack assay, in conjunction with pertinent clinical parameters, treatment response profiles, and MRD results from other assays (MFC, RT-PCR, and FISH) in real-world clinical practice.

Use of NGS to track clonal rearrangements of immunoglobulin and T-cell receptor genes is widely accepted as a valuable tool for MRD assessment [7, 11, 13]. NGS methodology has several notable advantages, including high sensitivity, broad applicability across diverse patient populations, elimination of the need for patient-specific oligonucleotide primers, and the ability to detect both oligoclonality and clonal evolution dynamics over time. It also offers the practical benefit of obviating the need for freshly acquired patient samples, in contrast to conventional methods such as flow cytometry [11–16]. In addition, NGS MRD has shown improvement in measurement precision, and prediction of relapse and survival outcomes of post-treatment MRD in pediatric B-ALL compared to MFC and PCR [11, 18, 19]. NGS MRD also enables the identification of new emerging clones during monitoring of relapsed ALL patients [20].

Nevertheless, there are several limitations to using the NGS clonality assay in patients with B-ALL. One important constraint is the potential for false positives and false negatives because of the assay’s exquisite sensitivity, which may detect minor clonal populations of uncertain clinical significance or miss rare variants. Furthermore, the phenomenon of pseudoclonality presents a challenge, as the emergence of novel clonal populations during treatment may introduce ambiguity regarding their clinical significance [21, 22]. This may be particularly problematic in samples obtained after intensive chemotherapy regimens when lymphocyte counts are diminished. The intricate nature of data analysis and interpretation poses further obstacles, demanding specialised expertise and resources for accurate assessment [22]. Moreover, full clonal heterogeneity may be difficult to detect in highly diversified leukaemia populations. The assay’s prolonged turnaround time and relatively high cost also warrant optimisation. Thus, while NGS holds substantial promise in MRD assessment, its limitations underscore the need to examine the correlation between NGS and other MRD assays in clinical studies and to refine its utility in clinical practice [7, 10, 11, 13, 23, 24].

The average turnaround time of LymphoTrack-MRD analysis included in this study was 11.1 days (range, 4 to 24 days). Despite the longer duration required for LymphoTrack-MRD analysis, its clinical implementation is not deemed problematic. This is because each assessment period for treatment response spans 4 to 8 weeks, and the early phase of intensification treatments exhibits uniformity across risk groups. Nonetheless, efforts should be directed towards reducing the longer turnaround time for early MRD measurement for SR patients in subsequent research.

An index clonal sequence was identified in 97.8% of our study cohort. MFC was used to assess disease status in all patients, and the concordance rate between MFC and LymphoTrack was 74.8%. The presence of positive clonality on high-throughput sequencing and negative results on MFC was previously found to be associated with worse outcomes than negative results on both tests [7, 11]. Our study did not statistically confirm these findings, however, when discordancy occurred, NGS+/MFC- was most common, and relapses occurred exclusively in NGS+/MFC- patients. Discordance can occur when MFC remission is achieved without LymphoTrack (possibly due to false-positive LymphoTrack results) or as an early sign of relapse. Svaton et al. reported that discordant cases between NGS MRD and PCR MRD for immunoglobulin/T cell receptor markers were enriched in low-specificity clonotypes with short junction lengths [18]. In our cohort, there was no significant difference in junction length between patients who experienced relapse and NGS+/MFC- patients who did not experience relapse (independent two-sample t-test, *P* = 0.4905 for *IGH* and *P* = 0.9757 for *IGK*).

Of the 58 patients lacking target markers for FISH or RT-PCR, 54 had clonality sequences allowing MRD monitoring. Concordance between RT-PCR and LymphoTrack was 70.7%. Furthermore, patients with sustained negative NGS clonality assay results had a favourable prognosis, highlighting its superior sensitivity and clinical utility as an assessment tool for assessing MRD, compared to MFC or RT-PCR [11, 18].

We also found that increased clone quantity or conversion to MRD positivity for *IGH* or *IGK* clonality was strongly associated with worse RFS, compared to decreased clone quantity or sustained negativity. These findings highlight the importance of vigilant monitoring of changes in NGS clonality for evaluating disease progression and therapeutic responses. They also suggest the need for more intensified treatment strategies to improve outcomes in patients manifesting an escalation in MRD status.

The 4 patients with bone marrow relapse exhibited clear evidence of elevated MRD levels of clone quantity or positive conversion on NGS clonality assay before morphologic relapse. Although pseudoclonal expansion is possible when lymphoid cell counts are low [25], the possibility of relapse must be seriously considered when new clone is detected. Notably, 2 patients displayed the emergence of new clonal sequences before morphologic relapse, and 1 patient exhibited a new *BCR::ABL1* mutation. Furthermore, in 1 patient initially presenting with 3 *IGH* clones, the least prevalent clone (16.64%) at diagnosis became the exclusive, dominant clone (90.28%) at relapse. These findings are suggestive of blast transformation [26] or expansion of a minor population of blasts during relapse [27]. Our results highlight the robust utility of the NGS clonality assay, which allows for early detection and monitoring of multiple productive gene rearrangements in ALL, especially in the context of MRD assessment.

Among relapsed patients, 2 patients (P35 and P50) showed positivity for *IGK*V3D-20-Kdel clonotypes. The manufacturer provided information on the low-specificity *IGK* clonotypes (Intron-Kdel, V3D-20 with any J or Kdel, and V3-11 with any J or Kdel) that may not be suitable for MRD analysis. However, in our patients, *IGK*V3D-20-Kdel clonotypes decreased or disappeared in CR and increased upon relapse, similar to other index clonotypes. Based on these experiences, we included the clonotype in our analysis. On the other hand, 4 patients in this study were positive only for the low-specificity clonotype and negative for MFC at CR (12 samples from 4 patients). These 4 patients did not relapse and maintained CR. Given that there are cases where low-specificity clonotypes are meaningful as an MRD marker and cases with the possibility of false positives, low-specificity clonotypes must be used in combination with other clonotypes from NGS and other MRD techniques such as RT-PCR and MFC.

This study had several limitations. First, we included all samples in the analysis without limiting minimal sequencing read count or input DNA amount; we did not apply the sensitivity threshold. Sequencing read count and input DNA amount determine the sensitivity of the LymphoTrack assay. In our analysis, 36.2% of the samples did not have sufficient sequencing read count and input DNA count to reach 10^− 4^ sensitivity with 95% confidence. Therefore, we must consider the possibility that the same level of sensitivity was not obtained in all samples, which affected the results of our analysis. However, our findings are meaningful in suggesting that significant MRD results can be obtained even with a level of assay performance that can be performed in real-world practice. Second, this study is its retrospective cohort design, which may have led to overestimated survival outcomes. Additionally, the treatment approach, including close monitoring and early consideration of HSCT for patients with persistent NGS clonality positivity, may have introduced bias. Furthermore, the short follow-up duration and limited number of patients precluded meaningful analysis of the impact of specific genetic mutations on survival outcomes.

In conclusion, this study highlights the potential of NGS clonality as a valuable tool for evaluating MRD in paediatric patients with B-ALL. Our findings demonstrated its ability to assess a larger number of patients than conventional methods and to predict disease deterioration before bone marrow relapse. Prospective studies with larger patient cohorts are required to explore tailored treatment strategies based on sustained MRD status. These efforts will contribute to advancing our understanding and management of paediatric ALL.

### Electronic supplementary material

Below is the link to the electronic supplementary material.

Supplementary Material 1

Supplementary Material 2

## Data Availability

No datasets were generated or analysed during the current study.
