# Supplementary material for: Monitoring measurable residual disease in paediatric acute lymphoblastic leukaemia using immunoglobulin gene clonality based on next-generation sequencing
Source: Cancer Cell Int. 2024 Jun 25;24:218. doi: 10.1186/s12935-024-03404-3 (PMC11201849; doi:10.1186/s12935-024-03404-3)

Figure S1. Treatment Scheme


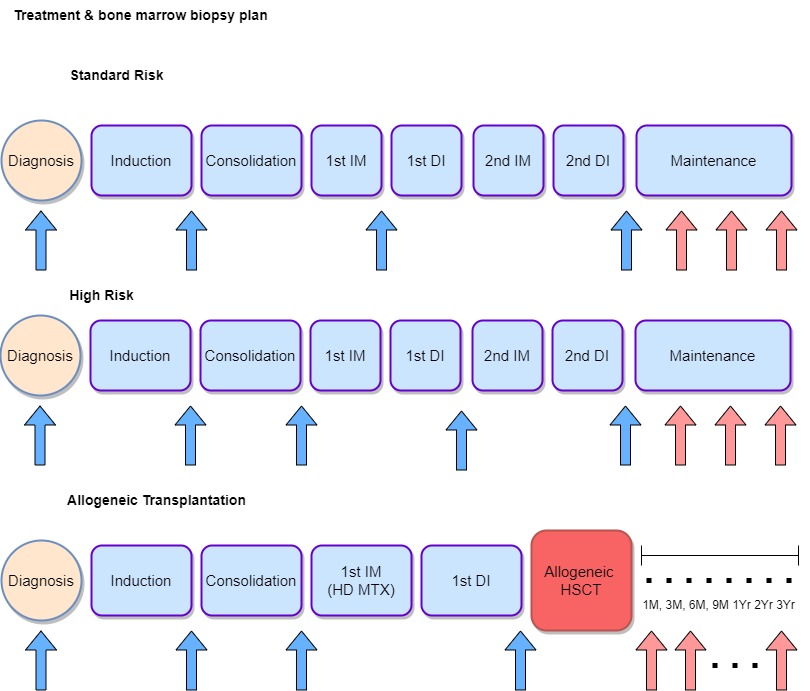


Two treatment regimens were utilized based on the patient's risk group, with pegylated asparaginase used alternatively to L-asparaginase. Bone marrow biopsies were performed at specified intervals during chemotherapy (blue arrows) and annually from the initiation of maintenance chemotherapy until the end of 3 years (red arrows). Patients who underwent transplantation followed the high-risk protocol, and bone marrow biopsies were conducted at 1 month, 3 months, 6 months, and 1 year post-transplantation, followed by annual assessments thereafter. Abbreviations: DI, delayed intensification; HSCT, hematopoietic stem cell transplantation.

1. **Standard-risk group**

Induction

Vincristine 1.5 mg/m^2^ on day (D) 2, 9, 16, 23

L-asparaginase 6,000 KU/m^2^ on D 3, 5, 7, 9, 11, 13, 15, 17, 19

Dexamethasone 6 mg/m^2^ on D 1–28

Triple intrathecal injection on D 1, 15 (methotrexate, hydrocortisone, cytarabine)

Consolidation

Cyclophosphamide 1,000 mg/m^2^ on D 1

Mercaptopurine 75 mg/m^2^ on D 1–28

Triple intrathecal chemotherapy on D 1, 8, 15, 22

First IM

Vincristine 1.5 mg/m^2^ on D 1, 11, 21, 31, 41

Methotrexate 100 mg/m^2^ on D 1

Methotrexate 150 mg/m^2^ on D 11

Methotrexate 200 mg/m^2^ on D 21

Methotrexate 250 mg/m^2^ on D 31

Methotrexate 300 mg/m^2^ on D 41

L-asparaginase 15,000 KU/m^2^ on D 2, 12, 22, 32, 42

Intrathecal methotrexate on D 2, 22

First delayed intensification

Vincristine 1.5 mg/m^2^ on D 1, 8, 15

Doxorubicin 25 mg/m^2^ on D 1, 8, 15

L-asparaginase 6,000 KU on D 2, 4, 6, 8, 10, 12

Dexamethasone 10 mg/m^2^ on D 1–7, 15–21

Cyclophosphamide 1,000 mg/m^2^ on D 29

Cytarabine 75 mg/m^2^ on D 29–32, 36–39

Mercaptopurine 75 mg/m^2^ on D 29–42

Triple intrathecal chemotherapy on D 33, 40

Second IM

Vincristine 1.5 mg/m^2^ on D 1, 11, 21

Methotrexate 200 mg/m^2^ on D 1

Methotrexate 250 mg/m^2^ on D 11

Methotrexate 300 mg/m^2^ on D 21

L-asparaginase 15,000 KU/m^2^ on D 2, 12, 22

Intrathecal methotrexate on D 3, 23

Second delayed intensification: same as first delayed intensification

Maintenance (males, 32 months; females, 22 months)

Vincristine 1.5 mg/m^2^ on D 1

Mercaptopurine 75 mg/m^2^ on D 1–28

Methotrexate (oral) 20 mg/m^2^ on D 1, 8, 15, 22

Prednisolone 40 mg/m^2^ on D 1–7

Intrathecal methotrexate every 3 months

1. **High-risk group**

Induction

Vincristine 1.5 mg/m^2^ on D 2, 9, 16, 23

Daunomycin 25 mg/m^2^ on D 2, 9, 16, 23

L-asparaginase 6,000 KU/m^2^ on D 3, 5, 7, 9, 11, 13, 15, 17, 19

Dexamethasone 10 mg/m^2^ on D 1–14 (changed to prednisolone on D 8 for patients aged >13 years)

Prednisolone 60 mg/m^2^ on D 15–28

Intrathecal cytarabine injection on D 1

Intrathecal methotrexate injection on D 8, 22

Consolidation

Cyclophosphamide 1,000 mg/m^2^ on D 1, 29

Cytarabine 75 mg on D 2–5, 9–12, 30–33, 37–40

Mercaptopurine 75 mg/m^2^ on D 1–14, 29–42

Vincristine 1.5 mg/m^2^ on D 15, 22, 43, 50

L-asparaginase 6,000 KU/m^2^ on D 15, 17, 19, 21, 23, 25, 43, 45, 47, 49, 51, 53

Intrathecal methotrexate on D 1, 15, 29, 36, 43, 50

First IM

Vincristine 1.5 mg/m^2^ on D 1, 15, 29, 43

Methotrexate 5,000 mg/m^2^ on D 1, 15, 29, 43

Mercaptopurine 25 mg/m^2^ on D 1–56

Intrathecal methotrexate on D 2, 30

First delayed intensification

Vincristine 1.5 mg/m^2^ on D 1, 8, 15

Doxorubicin 25 mg/m^2^ on D 1, 8, 15

L-asparaginase 6,000 KU on D 2, 4, 6, 8, 10, 12

Dexamethasone 10 mg/m^2^ on D 1–7, 15–21

Cyclophosphamide 1,000 mg/m^2^ on D 29

Cytarabine 75 mg/m^2^ on D 29–32, 36–39

Mercaptopurine 75 mg/m^2^ on D 29–42

Vincristine 1.5 mg/m^2^ on D 43, 50

L-asparaginase 6,000 KU/m^2^ on D 43, 45, 47, 49, 51, 53

Intrathecal methotrexate on D 3, 33, 40

Second IM

Vincristine 1.5 mg/m^2^ on D 1, 11, 21

Methotrexate 200 mg/m^2^ on D 1

Methotrexate 250 mg/m^2^ on D 11

Methotrexate 300 mg/m^2^ on D 21

L-asparaginase 15,000 KU/m^2^ on D 2, 12, 22

Intrathecal methotrexate on D 3, 23

Second delayed intensification: same as first delayed intensification

Maintenance (males, 32 months; females, 22 months)

Vincristine 1.5 mg/m^2^ on D 1

Mercaptopurine 75 mg/m^2^ on D 1–28

Methotrexate (oral) 20 mg/m^2^ on D 1, 8, 15, 22

Prednisolone 40 mg/m^2^ on D 1–7

Intrathecal methotrexate every 3 months

Figure S2. Distribution of *IGH* and *IGK* clones

The distribution of patients according to the number of *IGH* and *IGK* clones detected is shown. The bars indicate the number of patients of *IGH* and *IGK*, respectively.

Figure S3A. Correlation between *IGH* and multiparameter flow cytometry (MFC)


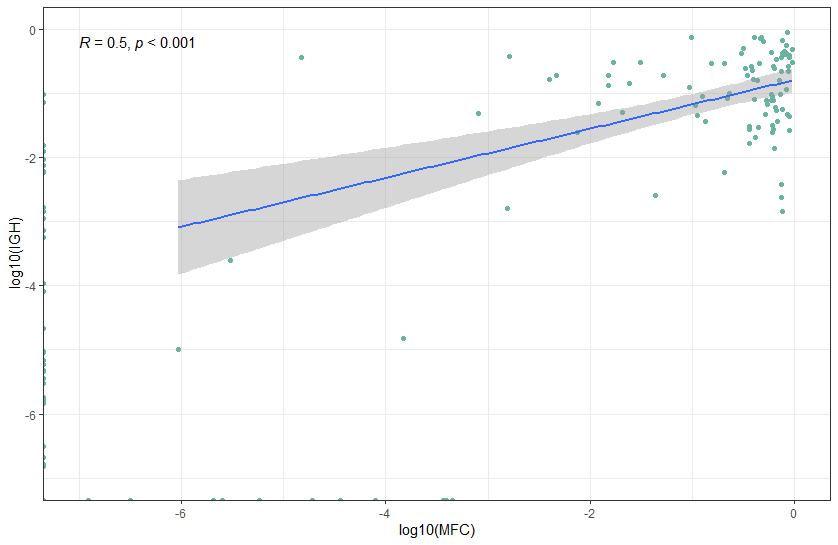


Figure S3B. Correlation between *IGK* and MFC


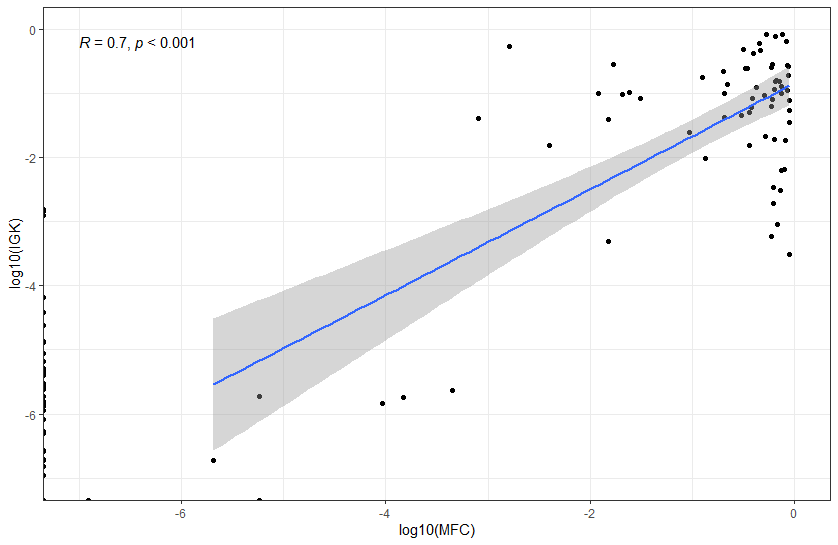


Concordance between *IGH*-MRD and multiparameter flow cytometry (MFC) and *IGK*-MRD and MFC. Specifically, it shows the correlation between MFC (x-axis) and *IGH*-MRD (y-axis) (3A) as well as the correlation between MFC (x-axis) and *IGK*-MRD (y-axis) (3B) across all bone marrow samples. Abbreviations: MFC, multiparameter flow cytometry; MRD, minimal residual disease

Figure S4. Relationships between positive measurable residual disease (MRD) status based on next-generation sequencing clonality and relapse-free survival (RFS). Patients with positive *IGH* NGS clonality results had significantly inferior RFS at both the first and second follow-up times (A, C). Patients with positivity of *IGK* alone had significantly inferior RFS at only the first follow-up time (B, D).


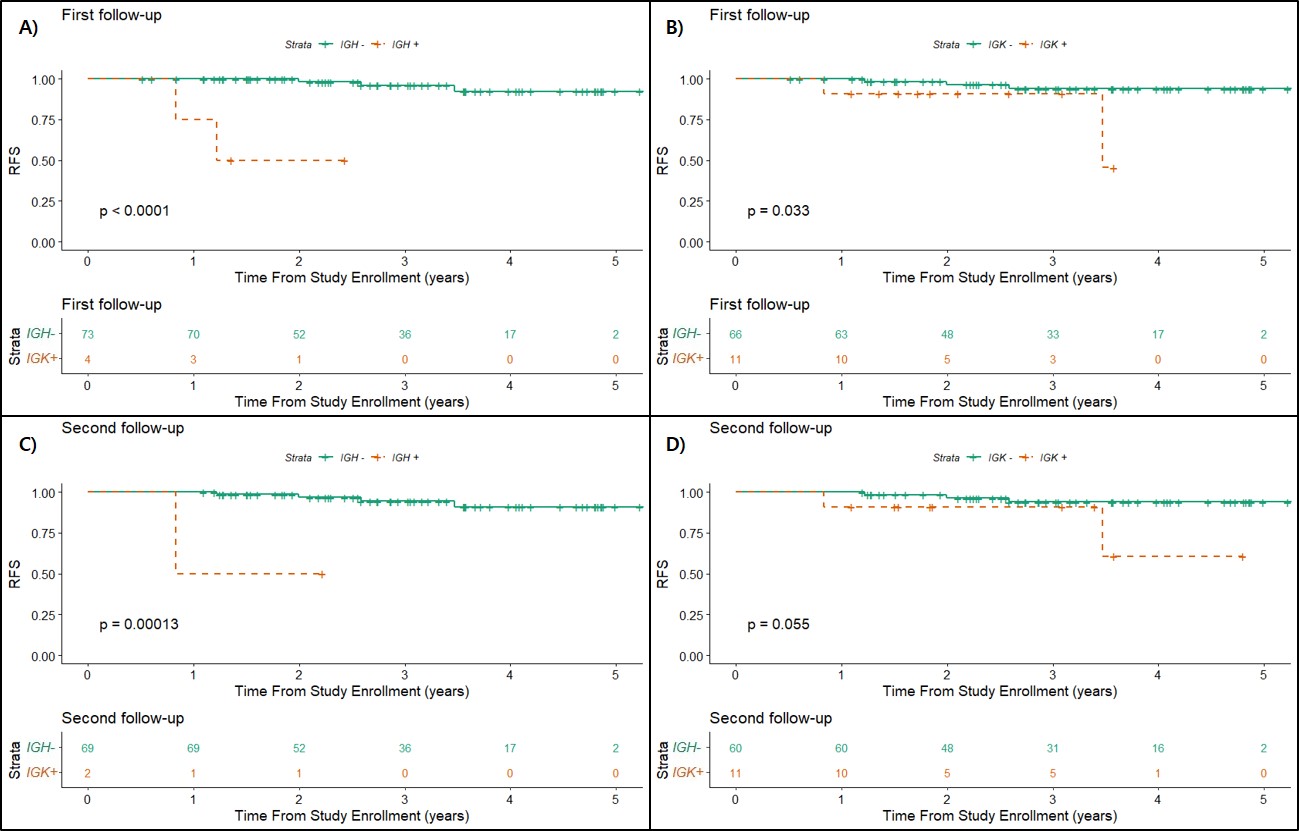


Figure S5. The changes of *IGH* and *IGK* clones in patients with bone marrow relapse. (A) P33: The third size *IGH* clone found at diagnosis becomes the dominant clone at the time of relapse. (B) P35: At diagnosis, two *IGH* clones and four *IGK* clones were identified. During the complete remission (CR), only some of them were detected, but upon relapse, the majority of the index clones were detected. (C) P92: At the time of diagnosis, two *IGH* clones and three *IGK* clones were detected, and upon recurrence, the diagnostic *IGH* index clones disappeared, and three *IGH* clones were newly observed. (D) P50: At relapse, new *IGK* clones were observed alongside the initial index *IGH* clone.


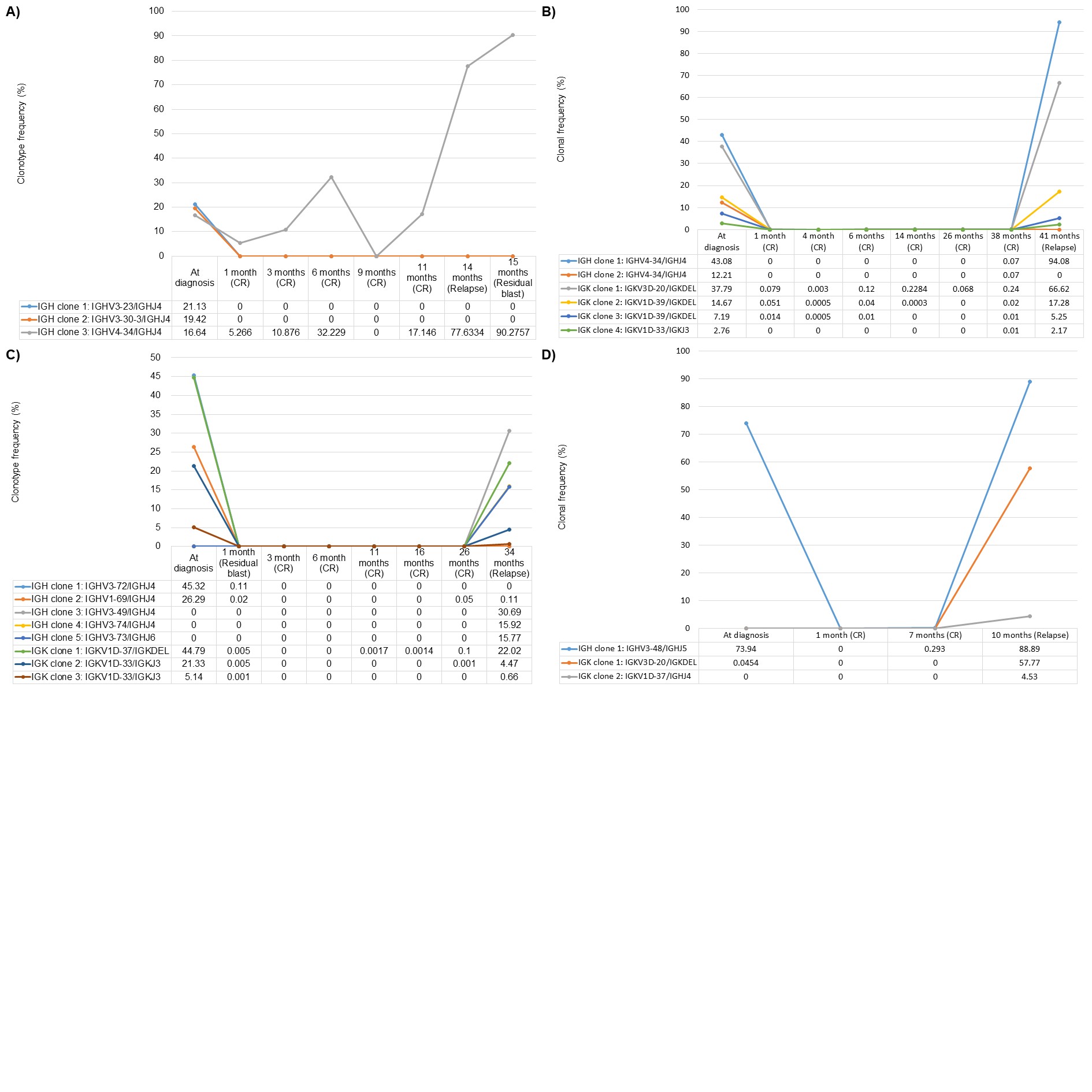

Supplement: Supplementary file 1 — Supplementary Material 1 [file 12935_2024_3404_MOESM1_ESM.docx]
