# Supplementary material for: Monitoring measurable residual disease in paediatric acute lymphoblastic leukaemia using immunoglobulin gene clonality based on next-generation sequencing
Source: Cancer Cell Int. 2024 Jun 25;24:218. doi: 10.1186/s12935-024-03404-3 (PMC11201849; doi:10.1186/s12935-024-03404-3)
Supplement: Supplementary file 2 — Supplementary Material 2 [file 12935_2024_3404_MOESM2_ESM.docx]

Table S1. Univariable and multivariable Cox regression analyses of factors associated with relapse

| **Univariable Cox Regression** | | | | |
| --- | --- | --- | --- | --- |
| **Factor** |  | **Hazard Ratio** | **95% CI** | ***P* value** |
| Age at diagnosis |  | 1.31 | 1.04–1.64 | 0.021 |
| Gene | *BCR::ABL1*-like | 10.44 | 1.70–64.09 | 0.011 |
|  | *TCF3::PBX1* | 25.52 | 2.28–286.20 | <0.01 |
| Elevated MRD levels of *IGH* |  | 23.56 | 2.44–227.20 | <0.01 |
| Elevated MRD levels of *IGH* or *IGK* |  | 9.62 | 1.05–88.06 | 0.045 |
| New clone(s) |  | 17.30 | 2.88–104.00 | <0.01 |
|  |  |  |  |  |
| **Multivariable Cox Regression** | | | | |
| **Factor** |  | **Hazard Ratio** | **95% CI** | ***P* value** |
| Age at diagnosis |  | 1.49 | 1.16–1.93 | <0.01 |
| Gene | *BCR::ABL1*-like | 142.70 | 17.20–1183.95 | <0.01 |
|  | *TCF3::PBX1* | 253.70 | 13.12–4906.19 | <0.01 |
| Elevated MRD levels of *IGH* or *IGK* |  | 36.05 | 2.98–436.23 | <0.01 |

Abbreviation: CI, confidence interval.
